# Normozoospermic men in infertile couples: Potential benefit of early medical diagnostic procedures

**DOI:** 10.1111/andr.70035

**Published:** 2025-03-19

**Authors:** Simone Bier, Anton Wolff, Michael Zitzmann, Sabine Kliesch

**Affiliations:** ^1^ Centre for Reproductive Medicine and Andrology University of Münster Münster Germany

**Keywords:** FSH‐B, hypogonadism, infertility, normozoospermic

## Abstract

**Introduction:**

Infertility, defined as the inability to achieve pregnancy despite regular, unprotected sexual intercourse for 1 year, affects approximately 15% of couples. Male factors contribute to 50% of these cases. The necessity of andrological evaluations for male partners of infertile couples with normozoospermia is currently under consideration.

**Methods:**

From 2010 to 2020, our center evaluated 997 patients presenting with infertility and normozoospermia. All patients underwent comprehensive assessments, including physical examinations, testicular sonography, blood tests, follice‐stimulating hormone beta (FSHB) c.‐211 variants, and semen analyses. For comparative purposes, we established two control groups: one comprising healthy men participating in the FAMe study (*n* = 201) and another consisting of men seeking fertility restoration following vasectomy (*n* = 75). Within the infertile male group, we further stratified patients into those with primary or secondary infertility.

**Results:**

Analysis of patient histories revealed a significantly elevated prevalence of genital malformations (e.g., hypospadias (*p* = 0.024) and undescended testes during childhood (*p* < 0.001) in our infertile group relative to the control group. By anamnesis we could find significant more patients with erectile dysfunction (*p* < 0.001) in our infertile men. The physical examination showed significant more patients with obesity in our infertility group (*p* < 0.001). Regarding hormonal profiles, a notably higher proportion of patients in the infertility group exhibited hypogonadism (*p* < 0.001), while compensated hypogonadism was more common in the control group. Reduced serum follicle‐stimulating hormone (FSH) concentrations in men with the FSHB c.‐211 GT/TT polymorphism versus the GG wildtype were only present in the infertile but not the fertile cohort (*p* < 0.001). Evaluation of ejaculate samples indicated a significant increase in round cells (*p* < 0.001) and leukocytes (*p* = 0.013) in our infertile patients compared to the healthy subjects.

**Discussion:**

The assessment of men presenting with infertility and normozoospermia unveiled a marked prevalence of physical and genetic findings. This underscores the critical need for andrological evaluations to prevent potential long‐term consequences.

**Conclusion:**

The andrological examination of normozoospermic and infertile men promises better health outcomes for the patients as well as it aids in refining fertility treatment options for their female counterparts.

## 1 INTRODUCTION

Infertility, as defined by the American Society of Reproductive Medicine, refers to the inability of a sexually active couple who are not using contraception to achieve a spontaneous pregnancy within a 12‐month period.^1^ The causes of infertility are nearly evenly distributed between males and females, with males contributing to 25%–39% and females to 33%–41% of cases. In some instances, both partners may contribute to 9%–39% of infertility cases.^2^

Guidelines from the Association of the Scientific Medical Societies in Germany (AWMF) recommend a comprehensive evaluation of infertile couples before considering assisted reproductive treatments. This evaluation includes both gynecological and andrological examinations.^2^ However, the AWMF guidelines do not currently recommend further andrological assessments once normozoospermia, as defined by specific reference values provided by the World Health Organization (WHO), is established. Similarly, the ESHRE guideline group recently published recommendations indicating no need for further evaluation of males with unexplained infertility when semen analysis according to WHO criteria is normal.^3^ These WHO reference values are based on the examination of semen from proven fathers and include one‐sided lower reference limits with a 95% confidence interval.^4^ It is important to note that the WHO explicitly states in its laboratory manual that even when semen values fall within the 95% confidence interval used to define normozoospermia, there is no guarantee of fertility.^5^

Previous research has shown a wide range of sperm characteristics even among fertile men.^6^, ^7^ Additionally, there is a significant overlap in sperm characteristics, such as sperm concentration and motility, between proven fertile men and men with infertility factors.^8^, ^9^, ^10^ Consequently, there is no clear‐cut threshold that can reliably distinguish fertile from infertile men.

Additional, the current literature recommended the follice‐stimulating hormone beta (FSHB) genotyping as diagnostic routine in patients with idiopathic infertility.^11^ This is due to the well‐established fact that an increased prevalence of the FSHB‐211 T allele is associated with lower follicle‐stimulating hormone (FSH) values in infertile men. Intact FSH production is crucial for adequate spermatogenesis.^12^, ^13^

Furthermore, it remains unclear whether the omission of andrological examinations in patients with normozoospermia and infertility leads to the oversight of abnormalities and pathologies that could have otherwise been diagnosed. For instance, there is existing evidence that infertility may be a risk factor for testicular germ cell tumors.^14^ About 30% of males with germ cell tumors are diagnosed while still revealing normozoospermia.^15^ Failing to diagnose such diseases could result in inaccurate counseling and, ultimately, overtreatment of the female partner. Therefore, this study aimed to assess the value of conducting further examinations in men diagnosed with normozoospermia and infertility to determine if such assessments can lead to earlier disease diagnoses, potentially allowing for effective treatment and avoiding unnecessary treatment of the female partner.

## 2 PATIENTS AND METHODS

The study received approval from the Institutional Review Board (Trial No. 2020‐945‐f‐S), and informed consent was obtained from each participating patient. We conducted a retrospective analysis by utilizing data from our institutional database, Androbase, as described by Tüttelmann et al. in 2006. We identified 997 patients who presented at our institute with infertility issues between 2010 and 2020, and a diagnosis of normozoospermia was established during their initial as well as in their second semen analysis in our laboratory, following the guidelines outlined in the 5th edition of the WHO manual actual for this period of time.^16^

To establish a control group (CG), we included two categories: first, 75 patients who sought fertility restoration after vasectomy between 2010 and 2020, and second, 201 healthy individuals who were recruited as part of our “Fertility and Ageing in Healthy Men (FAMe)” study, as reported by Laurentino et al. in 2020 (see Supporting information 3). Recruitment of healthy individuals for the ongoing “Fertility and Ageing in Healthy Men” study followed a rolling recruitment process. Only those healthy participants who had completed assessments of physical characteristics, semen parameters, and serum concentrations were included in the CG.^17^ The exclusion criteria for the FAMe study were as follows: smoking within the past year, use of illegal drugs, regular medication use (except for the treatment of mild hypertension, hypothyroidism, and dyslipoproteinemia), hospitalization within the past month, a history of or current cancer and cancer treatment, severe chronic renal failure, chronic viral infections, urogenital malformations or surgeries, prior diagnosis or treatment for infertility, chromosomal abnormalities, and participation in clinical trials within the past year.

All these patients underwent a comprehensive assessment, including a detailed medical history (anamnesis). Erectile function, loss of libido as well as ejaculate disorders were only assessed by anamnesis and not by validated questionnaires. We also performed physical examination, as well as sonographic evaluation of the testes and epididymis by using a high‐frequency 15‐MHz linear array scanner (ultrasound scanner ProFocus; BK Medical, Gentoft, Denmark). For calculation of the testis volume the ellipsoid method was used, in agreement with the current standards.^7^

Serum values of FSH, luteinizing hormone (LH), testosterone, sex hormone‐binding globulin (SHBG), estradiol, and prolactin were measured on an Abbot Architect i1000SR automated analyzer (Abbott Diagnostics) with the chemiluminescent microparticle immunoassay. Free testosterone was calculated from total testosterone and SHBG using the Vermeulen formula.

The ejaculate was analyzed according to the criteria specified in the 2010 WHO manual. All the results from these evaluations were included in our study.^16^

For genotyping DNA, extracted by using FlexiGene DNA extraction kit (QIAGEN, Düsseldorf, Germany), from EDTA‐preserved blood samples was used. It was performed with the StepOnePlus Real‐Time PCR‐System. Therefore, the TaqMan GTXpress Master Mix as well as the TaqMan genotyping assay mix, customized for the FSHB c.‐280 G > T single‐nucleotide polymorphisms (SNPs) was used.^12^

Furthermore, we categorized the men with normozoospermia and infertility into subgroups based on whether they had primary or secondary infertility.

For statistical analysis, we employed SPSS software (version 27.0). We used the Mann–Whitney *U*‐test to compare patient characteristics, blood parameters, and semen characteristics between groups. Regression analysis was performed to identify influencing factors of hypogonadism. The levene test for unequal variances was performed to evaluate the influence of the genotyping on FSH‐values in depending of the group.

## 3 RESULTS

### 3.1 Patient characteristics

At the time of their initial presentation, the patients with infertility were, on average, 35.8 years old, with a standard deviation of 5.7 years. In contrast, the healthy CG had an average age of 43.1 years with a higher standard deviation of 16.5 years, while patients seeking fertility restoration after vasectomy had an average age of 44.5 years with a standard deviation of 6.1 years. These age differences were statistically significant (*p* < 0.001).

### 3.2 Pre‐existing conditions

Specific andrological medical history revealed a significantly higher prevalence of erectile dysfunction (ED, *p* < 0.001), loss of libido (*p* = 0.011), and ejaculation disorders (*p* = 0.032) in the infertility group (IG).

Further medical history inquiries revealed a significantly higher incidence of genital malformations such as hypospadias in the IG compared to the CG (*p* = 0.024). Additionally, there were significantly more cases of undescended testes during childhood in the IG (*p* < 0.001) (see Table 1).

**TABLE 1:** Results of the specific andrological medical history.

	Infertil men *N* (%)	Men after vasectomy *N* (%)	Healthy probands *N* (%)	*p*‐value
Varicocele	190 (18.8%)	8 (10.4%)	64 (32.0%)	0.033
Maldescendet testis	112 (11.1%)	3 (3.9%)	2 (1.0%)	<0.001
Status post torsion of the spermatic cord	12 (1.2%)	2 (2.6%)	1 (0.5%)	0.999
Erectile dysfunction	30 (3.0%)	2 (2.6%)	0 (0.0%)	<0.001
Hypospadia	9 (0.9%)	0 (0.0%)	1 (0.5%)	0.024
Microlithiasis testis	73 (7.3%)	6 (8.1%)	9 (4.5%)	0.176
Loss of libido	15 (1.2)	0 (0.0%)	0 (0.0%)	0.011
Ejaculate disorders	9 (0.7%)	0 (0.0%)	0 (0.0%)	0.031

Within the context of our comprehensive medical history assessment, no significant differences were reported in the prevalence of cardiopulmonary diseases, thyroid gland diseases, gastrointestinal tract diseases, neurological diseases, or cancer diseases between the two groups (see Table 2).

**TABLE 2:** Pre‐excisting conditions.

	Infertil men *N* (%)	Men after vasectomy *N* (%)	Healthy probands *N* (%)	*p*‐value
Diabetes mellitus I	4 (0.4%)	2 (2.7%)	0 (0.0%)	0.479
Diabetes mellitus II	9 (0.9%)	1 (1.3%)	6 (3.0%)	0.820
Adiposity	150 (15.0%)	1 (1.3%)	18 (9.0%)	<0.001
Arterial hypertension	44 (4.4%)	3 (4.0%)	24 (11.9%)	0.281
Cardiopulmonary diseases	76 (7.6%)	3 (4.0%)	44 (21.9%)	0.534
Thyroid gland diseases	36 (3.6%)	0 (0.0%)	12 (6.0%)	0.798
Gastrointestinal tract diseases	23 (2.3%)	1 (1.3%)	13 (6.5%)	0.282
Neurological diseases	17 (1.7%)	1 (1.3%)	2 (1.0%)	0.494

### 3.3 Physical examination and sonography

All patients underwent measurements of weight and height, which allowed for the calculation of the body mass index (BMI). The IG exhibited a significantly higher prevalence of obesity (BMI > 30 kg m^2^) in comparison to the CG (*p* < 0.001) (see Table 2).

The volumetric analysis of the testes did not reveal a statistically significant difference between the IG (mean: 44.1 mL ± 13.0) and the CG (42.3 mL ± 13.3) (*p* = 0.099).

There were no significant differences observed in the incidence of testicular microlithiasis (*p* = 0.176), hydroceles (*p* = 0.072), spermatoceles (*p* = 0.295), or depending inhomogenic testicular lesions, independent of the kind of examination (clinical vs. ultrasound examination) (*p* = 0.434).

### 3.4 Serum concentrations of reproductive hormones

Examination of serum concentrations of reproductive hormones revealed several noteworthy differences between the IG and the CG. In the IG, we observed significantly lower levels of FSH (*p* = 0.003), LH (*p* < 0.001), total testosterone (*p* < 0.001), SHBG (*p* < 0.001), free testosterone (*p* < 0.001), and dihydrotestosterone (DHT) (*p* = 0.009). Conversely, estradiol and prolactin levels were significantly lower in the IG compared to the CG (*p* = 0.005 and *p* < 0.001). It is important to note that all patients fell within the normal range for these hormones (see Table 3).

**TABLE 3:** Hormonal levels of the different groups.

	Infertil men [mean ± SD]	Men after vasectomy [mean ± SD]	Healthy probands [mean ± SD]	*p*‐value
FSH‐value [U/L]	3.7 ± 2.1	4.8 ± 3.6	4.0 ± 3.2	0.003
LH‐Value [U/L]	2.9 ± 1.4	3.4 ± 1.8	3.2 ± 1.3	<0.001
Total testosterone [nmol/L]	16.5 ± 6.5	16.6 ± 5.3	22.0 ± 7.0	<0.001
Free testosterone [nmol/L]	345.6 + 124.9	302.7 + 77.9	432.1 + 128.7	<0.001
SHBG [nmol/L]	34.0 + 14.5	39.7 + 10.1	40.6 + 15.8	<0.001
DHT [nmol/L]	0.78 ± 0.35	0.83 ± 0.16 (*n* = 3)	0.83 ± 0.28	0.009
Prolactin [mU/L]	190.9 + 274.1	162.3 + 79.6	230.1 + 141.2	<0.001
Estradiol [pmol/L]	82.2 + 29.8	74.8 + 26.6	87.9 + 27.7	0.005

Abbreviations: DHT, dihydrotestosterone; FSH, follice‐stimulating hormone; LH, luteinizing hormone; SHBG, sex hormone‐binding globulin.

When evaluating patients with hormonal values outside the normal range, we found a significantly higher number of patients with decreased LH values in the IG (*p* < 0.001) and a significantly higher number of patients with increased FSH values in the control cohort (*p* = 0.01). Regarding hypogonadism, defined as a total testosterone value of < 12 nmol/L, we identified a significantly higher number of patients in the IG with hypogonadism (*p* < 0.001). Conversely, in the CG, there were significantly more patients with compensated hypogonadism, defined as having a total testosterone value of ≥12 nmol/l with increased LH and/or FSH levels (*p* = 0.005) (see Table 4).

**TABLE 4:** Decompensated and compensated hypogonadism.

	Infertil men *N* (%)	Men after vasectomy *N* (%)	Healthy probands *N* (%)	*p*‐value
Increased FSH (> 7 U/L)	55 (5.5%)	10 (13.3%)	17 (8.5%)	0.010
Decompensated hypogonadism (total testosterone < 12 nmol/L)	257 (25.8%)	15 (20.0%)	13 (6.5%)	0.001
Compensated hypogonadsim (total testosterone in the normale range, increased LH/FSH‐values)	46 (4.6%)	9 (12.0%)	16 (8.0%)	**0.005**

Abbreviations: FSH, follice‐stimulating hormone; LH, luteinizing hormone.

Additionally, decompensated hypogonadism was significant associated with a higher BMI‐score (*p* < 0.001).

We also evaluated the odd ratio (OR) for hypogonadism. The OR for hypogonadism in our NIG was 2.6 (95% confidence interval 1.7–4.1), when it was adjusted by BMI. If it was not adjusted by BMI, the OR for hypogonadism was 3.0 (95% confidence interval 2.1–4.6). There was no association with age.

### 3.5 Follice‐stimulating hormone beta‐211 genotype

In 832 patients of the NIG as well as in 234 patients of the CG, the SNP c.‐211G > T was examined. In the NIG, 619 (74%) had the genotype FSHB c.211 GG, 196 (*n* = 24%) the genotype FSHB c.211 GT and 2% (*n* = 17) the genotype FSHB c.211 TT. In our CG, we found 167 (71%) with the GG type, 58 (25%) with the GT type, and 9 patients (4%) with the TT‐type. The FSH‐value in infertile men the FSH‐value was 3.8 ± 2.2 when they had the genotype FSHB c.211GG, and it was significant lower, then they had the genotype FSHB c.211GT/TT (3.1 ± 1.6, *p* < 0.001). Among men with the genotype FSHB c.‐211 GG, there was no significant difference in FSH values between the infertile (3.8 ± 2.2) and fertile men (4.0 ± 3.2) (*p* = 0.48). However, in men with the genotype FSHB c.‐211 GT/TT, a significantly higher FSH value was observed in fertile men (4.2 ± 2.5) compared to infertile men (3.1 ± 1.6) (*p* < 0.001).

Subgroup analysis of the infertile men revealed that the FSH value was significantly lower in those with the genotype FSHB c.‐211 GT/TT compared to those with the genotype FSHB c.‐211 GG (*p* < 0.001). In the group of fertile men, there was no significant difference in FSH values between those with the genotype FSHB c.‐211 GG and those with the genotype FSHB c.‐211 GT/TT (*p* = 0.69).

### 3.6 Semen analysis

The CG for semen analysis comprised exclusively healthy participants from the FAMe study, as assessing the semen of patients after vasectomy was not feasible. Semen analysis results indicated significant differences between the IG and the CG.

In the IG, there was a significantly higher sperm concentration (*p* < 0.001) and a greater total sperm count (*p* < 0.001) in the ejaculate, while the semen volume did not exhibit a significant difference between the two groups (*p* = 0.093). Moreover, the morphology assessment revealed a significantly higher proportion of normally shaped spermatozoa in the IG (*p* < 0.001). Conversely, the CG demonstrated a significantly better AB‐motility compared to the IG (*p* = 0.003).

Biochemical markers in the seminal plasma indicated no significant difference in glucosidase levels; however, zinc levels were significantly higher in the IG (*p* = 0.028), and fructose levels were significantly elevated as well (*p* < 0.001). The semen of infertile men also exhibited a significantly higher presence of round cells (*p* < 0.001) and leukocytes (*p* = 0.013). Moreover, the MAR‐Test (IgG) showed a significantly higher frequency of positive results in the IG (*p* = 0.040), while no difference was observed in the MAR‐Test (IgA).

### 3.7 Sub‐group analysis—primary infertility versus secondary infertility

In the subgroup analysis, we further divided the IG into patients with primary infertility (*n* = 837) and patients with secondary infertility (*n* = 160). Notably, the patients with secondary infertility were older, with an average age of 39.5 years and a standard deviation of 5.9 years. In contrast, the men with primary infertility were significantly younger (*p* < 0.001), with an average age of 35.1 years and a standard deviation of 5.4 years.

Within this subgroup, semen analysis results showed no significant difference in semen volume (*p* = 0.165), the number of spermatozoa (*p* = 0.065), AB‐motility (*p* = 0.554), or sperm morphology (*p* = 0.645) between the two groups. However, the concentration of spermatozoa (*p* = 0.005), A‐motility (*p* = 0.045), and glucosidase levels (*p* = 0.048) were significantly higher in the group of patients with secondary infertility (see Table 5).

**TABLE 5:** Semen analysis of the subgroup: primary versus secondary infertility.

	Patients with primary infertility [mean ± SD]	Patients with secondary infertility [mean ± SD]	*p*‐value
Volume [mL]	3.6 ± 1.7	3.8 ± 1.7	0.165
Concentration [Mio/mL]	69.6 ± 59.7	59.7 ± 45.9	0.005
Sperm count [Mio]	227.8 ± 182.1	200.0 ± 150.2	0.065
AB‐Motility [%]	51.1 ± 7.4	50.5 ± 6.7	0.554
A‐Motility [%]	39.0 ± 8.3	37.4 ± 8.0	0.045
B‐Motility [%]	12.1 ± 5.8	13.1 ± 5.6	0.008
Morphology [%]	5.4 ± 1.5	5.5 ± 1.5	0.645

Regarding serum concentrations, there was no significant difference in total testosterone values between the two groups (*p* = 0.178). However, the FSH value was significantly higher in the group of proven fathers (*p* = 0.020), while the LH value was significantly higher in the patients with primary infertility (*p* = 0.040).

## 4 DISCUSSION

In this study, we conducted an assessment of andrological history and examinations in men with normozoospermia and infertility within couples. Starting with the medical history, the patients in the IG reported significantly higher rates of ED, loss of libido, and ejaculation disorders compared to our CG These findings are consistent with prior research that has linked ED, depressive symptoms, and dysfunctional sexual relationships with individuals experiencing couple infertility.^18^ In one of six men in infertile men ED and/or premature ejaculation could be expected.^19^ Lotti et al. could even show a worsening of the erectile function with a worsening of the semen quality and in line with our results more often ED in normozoospermic infertile men compared to fertile men.^20^ Notably, prior studies have established a significant relationship between ED and depression in men with couple infertility, while one study has ruled out a connection between spermatogenesis and ED in such patients.^21^ These previously published findings align with our medical history results, emphasizing the psychological stress associated with couple infertility. However, the erectile function and loss of libido was only assessed by anamnesis in this study. The use of validated questionnaires would help to objectify the results and made them more comparable.

Further medical history inquiries revealed a higher prevalence of hypospadias in the IG compared to the CG. This observation aligns with various studies that have demonstrated a significant impact on fertility in males with hypospadias.^22^,^23^ However, it remains a subject of debate whether hypospadias is associated with impaired semen parameters. Asklund et al. reported impaired semen parameters in patients with hypospadias when accompanied by additional genital disorders, whereas patients with isolated hypospadias did not show limitations. Conversely, Kumar et al. showed that semen restrictions could be detected in patients with proximal types of hypospadias, while those with distal types did not exhibit any semen quality restrictions.^23^,^24^ It is important to note that our study had only nine patients with hypospadias in the IG and one patient with hypospadias in the CG, which calls for further investigation to assess the impact of hypospadias on fertility more comprehensively.

While the total testosterone value, free testosterone value, and DHT were significantly higher in our IG compared to the CG, we observed a higher prevalence of hypogonadism in the IG. Even though the CG was significantly older than the IG, we found a significantly higher number of men with compensated hypogonadism in the CG compared to the IG. Notably, the IG also had a higher prevalence of obesity compared to the CG and also in our study cohort, hypogonadism was significant associated with a higher BMI.

These findings align with current literature indicating a well‐established association between limited semen parameters and hypogonadism. Additionally, hypogonadal men have been documented to have a connection with obesity, increased waist circumference, higher systolic blood pressure, metabolic syndrome, and osteoporosis. Consequently, it is understandable that hypogonadal men require further medical treatment to mitigate long‐term negative effects. However, the novel insight from this study is that even in patients with normozoospermia in infertile couples, there is an elevated risk for hypogonadism. This implies that hypogonadal men, even those with normozoospermia, are at risk for excess morbidity and mortality, as corroborated by previous research.^25^, ^26^,^27^

Interestingly, we see significant lower gonadotropins in our NIG compared to the CG, although there was significant more often an undescended testis documented, which normally comes along with higher gonadotropins. It is well known that the metabolic syndrome is well associated with hypogonadism, poor sperm morphology, ED, and depression.^28^ However, if there is a dependence between metabolic syndrome and low gonadotropins is still discussed.^29^Moreover, the study indicates a significant higher risk for ED in men from infertile couples, but it did not establish a clear relationship between ED and serum concentrations, as demonstrated by Lotti et al. in 2012.^18^

Although the analysis of the FSHB genotype is no standard in the routine, we decided to evaluate it. In line with current literature, we observed a significantly lower FSH value in the IG among men with the genotype FSHB c.‐211 GT/TT compared to those with the FSHB c.‐211 GG genotype.^13^, ^30^ Interestingly, there was no difference in FSH values in the CG regardless of genotype. In the subgroup analysis of men with the genotype FSHB c.‐211 GT/TT, the FSH value was significantly higher in the CG compared to the IG.

Fertile men appear to compensate for the lower transcriptional activity associated with the GT/TT genotype, leading to regular serum concentrations of FSH. Conversely, infertile men with the GT/TT genotype present with lower FSH levels than those with the GG wildtype. It is speculated that these infertile men with the GT or TT genotype might benefit from the external administration of recombinant FSH. Smaller studies have shown positive results when men with the GT or TT genotype were treated with recombinant FSH, but larger, placebo‐controlled studies are needed to confirm this. ^11^, ^31^, ^32^, ^33^

The selection of patients for our study involved a retrospective examination of all individuals with infertility, specifically targeting those who met the criteria for normozoospermia. As a result, it is not surprising that our evaluation of semen parameters revealed significantly higher numbers of spermatozoa, a better concentration, and improved morphology in the semen analysis of our IG compared to our CG. However, our CG exhibited significantly better A+B‐motility, and we also observed a significantly higher presence of round cells in the semen of our male patients with infertility.

The presence of round cells, particularly leukocytes (white blood cells), can be indicative of urogenital infections.^34^ Assessing these cells in semen can be challenging. Existing literature provides contradictory findings, with some studies showing no correlation between fertility and leukocytospermia, while others suggest that leukocytospermia can either improve sperm quality or lead to an improved pregnancy rate.^34^

Regardless of these differing viewpoints, the observed increase in round cells in the semen of our patients in the IG is a verified finding, and it cannot be ruled out that this may have an effect on the pregnancy rate. Further research may be necessary to elucidate the impact of round cells, particularly leukocytes, in semen on fertility and pregnancy outcomes.

One limitation of the study is the missing of the transrectal ultrasound (TRUS), in particular we found significant more round cells and leucocytes in the ejaculate of our NIG. It is well known that TRUS enables the imaging of the prostate as well as the seminal vesicles (SV) and it can be used to assess prostate inflammation.^35^ So, especially patients with infections can benefit from an additional examination.

It would indeed be highly informative to investigate female factors and the specific treatments the couples underwent. Unfortunately, we were unable to retrospectively evaluate the female factors in these couples. One key reason for this limitation is that we collaborate with multiple fertility centers, making it challenging to obtain consistent and comprehensive data on the female partners. This is definitely are marked limitation of our paper.

## 5 CONCLUSION AND SUMMARY

The comprehensive evaluation of male patients with normozoospermia and couple infertility has revealed a spectrum of notable medical issues. Some of these issues have been already well‐documented in patients with impaired semen parameters, some are associated with higher morbidity and mortality. Consequently, a thorough medical examination of patients with infertility, even when normozoospermia is confirmed, is strongly recommended. This approach can serve to prevent adverse long‐term consequences which are associated with hypogonadism and ensure more accurate diagnosis and effective treatment.

## AUTHOR CONTRIBUTIONS

**Simone Bier**: Design; data evaluation+statistics; writing. **Anton Wolff**: Design, data evaluation+statistics; **Michael Zitzmann**: Data evaluation+statistics; writing. **Sabine Kliesch**: Design, writing.

## Supporting information



Supporting information
